# Parkinson's disease and atypical parkinsonism: the importance of magnetic resonance imaging as a potential biomarker

**DOI:** 10.1590/0100-3984.2017.50.4e1

**Published:** 2017

**Authors:** Henrique Carrete Jr.

**Affiliations:** 1 Adjunct Professor in the Department of Diagnostic Imaging of the Escola Paulista de Medicina da Universidade Federal de São Paulo (EPM-Unifesp), São Paulo, SP, Brazil. E-mail: hcarrete@gmail.com.

Parkinson's disease is one of the most common neurodegenerative diseases, characterized by motor and non-motor manifestations, which mainly affects the elderly but can also occur in other age groups^(1)^. The disease results from a specific process of degeneration of neurons in the substantia nigra pars compacta, located in the midbrain, with a consequent loss of function of the nigrostriatal dopaminergic pathway, leading to a progressive reduction in dopaminergic neurotransmission to the striatum, especially to the putamen. Although the cause of that degeneration is not yet fully understood, there is an apparent correlation with increased iron deposition in the substantia nigra or with a local reduction in the quantity of neuromelanin, an iron chelator considered neuroprotective^(2-4)^. The definitive diagnosis of Parkinson's disease requires a histological finding of intraneuronal inclusions (Lewy bodies) in the substantia nigra.

The clinical manifestations of Parkinson's disease include bradykinesia, stiffness, resting tremor, and postural instability^(5)^. Other neurodegenerative diseases can present this clinical syndrome (parkinsonism), thus mimicking Parkinson's disease. Such diseases include Lewy body dementia, progressive supranuclear palsy, multiple system atrophy, and corticobasal degeneration, typically referred to, collectively, as atypical parkinsonism. Therefore, Parkinson's disease presents a variable clinical spectrum and overlaps with multiple neurological conditions of various causes, making it one of the most difficult diseases to diagnose, particularly in its early stages, resulting in treatment delays or even inappropriate treatment. Because of this overlap of signs and symptoms, together with the lack of any biological markers to differentiate these conditions, diagnostic errors occur in up to 25% of cases, even at centers specializing in movement disorders^(6)^.

In this scenario, it is of paramount importance to combine the clinical evaluation with adjuvant diagnostic methods. Those that have advanced the most in recent years are functional and structural imaging tests, including positron-emission tomography, single-photon emission computed tomography, and conventional magnetic resonance imaging (MRI).

In individuals with Parkinson's disease, conventional MRI sequences, including T1-weighted, T2-weighted, T2-weighted fluid-attenuated inversion recovery, and proton density-weighted sequences, typically show no abnormalities or only aging-related changes. Certain findings, such as putaminal atrophy in multiple system atrophy, have high specificity for the diagnosis of other parkinsonian syndromes, although such findings have low sensitivity, especially in the early stages of the disease^(7)^.

The evolution of MRI imaging methods has improved the ability to detect alterations characteristic of Parkinson's disease, as well as to differentiate between Parkinson's disease and other parkinsonian syndromes. In recent years, MRI has revealed several potential biomarkers that could provide important information about the disease and, it is hoped, detect early neuropathological findings and mechanisms of adjacent neurodegeneration, as well as correlating with the progression of the disease, thus allowing its status to be monitored^(8)^. Such information also allows, among other things, a quantitative assessment of typical Parkinson's disease alterations by means of estimates of biochemical measurements, tissue volume, and the macrostructural/microstructural integrity of the brain tissue affected, particularly in the substantia nigra and basal nuclei. For example, proton or phosphorus magnetic resonance spectroscopy enables estimates of concentrations and of energy metabolism in affected segments. Currently, there are also a number of sequences for the evaluation of iron deposition in nerve tissue, quantitative maps of susceptibility being the most important for estimating the level of local neuronal degeneration. Through the analysis of volumetric sequences, either manually or with any of a variety of specific types of software, local parenchymal atrophy in the affected areas can be estimated. Diffusion-weighted imaging and diffusion tensor imaging sequences provide several indices that characterize the motion of molecules (expressed as apparent diffusion coefficients and mean diffusivity), the orientation of the diffusion with fractional anisotropy, and the characteristics of the diffusion along and perpendicular to its principal direction (axial or longitudinal), making it possible to evaluate tissue integrity^(9-11)^.

The excellent review article by Oliveira et al.^(12)^ published in this issue of **Radiologia Brasileira**, addresses precisely the value of MRI diffusion techniques in the evaluation of Parkinson's disease and in its differential diagnosis with atypical parkinsonism. That review demonstrates the true potential of those techniques to reveal biomarkers of this important neurodegenerative disease, the prevalence of which is on the rise in the context of the aging of the world's population.
